# Deciphering pathogenicity and virulence of the first *Staphylococcus debuckii* isolate from diabetic foot osteomyelitis

**DOI:** 10.3389/fcimb.2024.1489280

**Published:** 2024-12-17

**Authors:** Cynthia N. Abi Najem, Chloé Magnan, Lucile Plumet, Nour Ahmad-Mansour, Cassandra Pouget, Madjid Morsli, Alix Pantel, Karima Kissa, Albert Sotto, Jean-Philippe Lavigne, Virginie Molle

**Affiliations:** ^1^ VBIC, INSERM U1047, University of Montpellier, Montpellier, France; ^2^ VBIC, INSERM U1047, University of Montpellier, Department of Microbiology and Hospital Hygiene, CHU Nîmes, Nîmes, France; ^3^ VBIC, INSERM U1047, University of Montpellier, Department of Infectious Diseases, CHU Nîmes, Nîmes, France

**Keywords:** *Staphylococcus debuckii*, diabetic foot osteomyelitis, coagulase-negative staphylococci, biofilm formation, intracellular survival, zebrafish embryo model, pathogenicity, virulence

## Abstract

**Introduction:**

This study identifies *Staphylococcus debuckii* as a new coagulase-negative staphylococcal species isolated from diabetic foot osteomyelitis (DFOM) and provides an in-depth analysis of its pathogenic and virulence profile, as well as demonstrating its potential to cause infection.

**Methods:**

The *S. debuckii* NSD001 strain was examined for its planktonic growth, biofilm production, and phagocytosis rates in murine macrophages compared to *S. aureus* NSA739. Additionally, persistence and replication within human osteoblasts were investigated, while the zebrafish embryo model was employed to assess virulence. Genomic sequencing and bioinformatic analysis were also conducted to identify genes associated with virulent potential.

**Results and Discussion:**

*S. debuckii* NSD001 exhibited robust planktonic growth and significant biofilm production, highlighting its capacity to initiate and maintain an infection, and demonstrated similar rates of phagocytosis as *S. aureus* NSA739 in murine macrophages, suggesting a mechanism for evading initial host defenses. The strain persisted and replicated within human osteoblasts, indicative of a strategy for intracellular survival and facilitation of chronic osteomyelitis. The zebrafish embryo model revealed a slower, yet fatal, virulence profile for *S. debuckii* NSD001 compared to the rapid lethality induced by *S. aureus* NSA739. Genomic sequencing and bioinformatic analysis uncovered various genes corroborating its virulence. *S. debuckii* NSD001 poses a significant concern in DFOM due to its ability to form biofilms and survive within host cells, presenting challenges for current treatment strategies. This underscores the need for updated clinical protocols and increased awareness among healthcare professionals to effectively manage infections caused by this emerging pathogen.

## Introduction

Diabetic foot ulcers (DFUs) constitute a prevalent, deleterious, and costly complication of diabetes mellitus, affecting one third of all diabetic individuals during their lifetime (Armstrong et al., 2017). The primary risk factors for DFU development include peripheral neuropathy, peripheral arterial disease, and repetitive trauma to the foot. It has been established that infections, which affect over half of all DFUs, can progress to involve both soft and osseous tissues, potentially culminating in diabetic foot osteomyelitis (DFOM) (Lavery et al., 2006; Prompers et al., 2007). These complications notably contribute to a diminished quality of life, the necessity for varying degrees of amputation, and increased morbidity (Senneville et al., 2023). While the infectious agents in DFUs and DFOM are typically polymicrobial, there is a notable predominance of Gram-positive cocci, specifically *Staphylococcus aureus* and coagulase-negative staphylococci (CoNS) (Senneville et al., 2006; Jneid et al., 2018). Traditionally, CoNS are considered less pathogenic compared to *S. aureus*. Nevertheless, CoNS species have been increasingly implicated in severe infections, bearing substantial clinical relevance, not only in immunocompromised patients but also in healthy individuals (Michels et al., 2021). In DFOM, *Staphylococcus epidermidis* is commonly isolated (Aragón-Sánchez et al., 2010), with less frequent cases involving *Staphylococcus pettenkoferi* (Loïez et al., 2007; Ahmad-Mansour et al., 2021; Magnan et al., 2022), *Staphylococcus lugdunensis* (Kear et al., 2016), and *Staphylococcus schleiferi* (Nguyen et al., 2020). In this report, we describe the first known case of DFOM attributed to *Staphylococcus debuckii*. The first strain, SDB 2975^T^, was isolated from bovine milk in 2007 and originated from a dairy herd in Quebec, Canada. It was not until 2019 that Naushad et al. recognized and described these isolates as a new *Staphylococcus* species, *S. debuckii* sp. nov (Naushad et al., 2019). This CoNS subgroup is typically associated with food products, mammalian skin, and occasionally with infections linked to implanted medical devices. However, until now, these CoNS had not been reported as pathogens in humans (Becker et al., 2020). Therefore, the emergence of *S. debuckii* presents a novel clinical challenge and this study aims to delineate the pathogenic profile of a clinical *S. debuckii* isolate from a DFOM patient.

## Materials and methods

### Bacterial strains, media and growth conditions

*S. debuckii* NSD001, the studied strain, and *S. aureus* NSA739, a reference strain from our team, were isolated from patients with DFOM at Nîmes University Hospital, France. The *S. debuckii* isolate was found in four out of five samples, including two tissue biopsies and two bone biopsies on the healthy bone. The strain demonstrated susceptibility to all tested antibiotics, notably to glycopeptides (with MICs of 0.5 mg/L for vancomycin and teicoplanin, and 0.06 mg/L for dalbavancin) and advanced generation cephalosporins (with MICs of 0.125 mg/L for ceftaroline and 0.5 mg/L for ceftobiprole). Bacterial identification was performed by mass spectrometry using Vitek-MS^®^ (Biomérieux, Marcy-l’Étoile, France) and labeled as *S. debuckii* NSD001. Antimicrobial susceptibility testing (Penicillin G 1U, cefoxitin 30 µg, erythromycin 15 µg, clindamycin 2 µg, quinupristin-dalfopristin 15 µg, kanamycin 30 µg, tobramycin 10 µg, gentamicin 10 µg, minocycline 30 µg, ofloxacin 5 µg, fusidic acid 10 µg, fosfomycin 200 µg, rifampicin 5 µg, cotrimoxazole 25 µg, linezolid 10 µg) of these isolates was performed by disk diffusion test (Bio-Rad, Marnes-La-Coquette, France) on Mueller-Hinton (Bio-Rad, Marnes-La-Coquette, France) agar plates according to European Committee for Antimicrobial Susceptibility Testing (EUCAST 2024) recommendations (https://www.eucast.org/clinical_breakpoints). Dalbavancin, ceftaroline and ceftobiprole MICs were determined by MIC Test Strips (Liofilchem, Roseto degli Abruzzi, Italy and BioMérieux). Daptomycin, vancomycin and teicoplanin MICs were determined using broth microdilution procedures (UMIC) (Bruker Daltonics, Champs sur Marne, France). Antibiotic susceptibility was interpreted using the EUCAST breakpoints. *Staphylococcus* strains were cultivated on Tryptic Soy Agar (TSA) or grown in Tryptic Soy Broth (TSB) medium at 37°C and 180 rpm. Bacterial growth curves were monitored using a microplate reader (Tecan, Model Spark, Grödig, Austria GmbH) at an optical density of 600 nm (OD_600_ nm) for 24 h at 37°C and 108 rpm.

### Macrophage culture and infection

The murine macrophage cell line RAW 264.7 (mouse leukemic monocyte macrophage, ATCC TIB-71) was grown in Dulbecco Modified Eagle Medium (DMEM) (Thermo Fisher Scientific) supplemented with 10% fetal calf serum (Thermo Fisher Scientific) at 37°C in a humidified atmosphere with 5% CO_2_. *S. debuckii* NSD001 and *S. aureus* NSA739 strains were grown to the mid-exponential growth phase (OD_600_ nm = 0.6–0.8) in TSB medium for macrophage infection. After 5 min at 3220 rpm, the collected bacteria were resuspended in sterile phosphate-buffered saline (PBS). RAW 264.7 cells (5x10^5^ cells/mL in 24-well plates) were infected with *S. debuckii* NSD001 or *S. aureus* NSA739 at a multiplicity of infection (MOI) of 20:1 (bacteria/cells) for 1 h at 37°C and 5% CO_2_. After infection, the extracellular bacteria were then killed by adding 100 µg/mL of gentamicin for 30 min. Subsequently, macrophages were washed twice with PBS (T0) and then incubated for 5 and 24 h in fresh medium with 5 μg/mL of lysostaphin. Intracellular bacteria were counted at times 0, 5 and 24 h by lysing infected macrophages with 0.1% Triton X-100 in PBS. The number of colony-forming units (CFU) was determined by plating macrophage lysates, serially diluted in 1X PBS, onto TSA plates, with three biological replicates, each performed in triplicate, and plates were incubated for 24 h at 37°C.

### Osteoblast culture and infection

The human osteoblast cell line MG63 (Billiau et al., 1977) was cultured in DMEM (Thermo Fisher Scientific) supplemented with 10% fetal calf serum (Thermo Fisher Scientific), 1% Glutamate (Gibco™) and 0.1% penicillin/streptomycin antibiotics (Gibco™) at 37°C in a humidified atmosphere with 5% CO_2_. *S. debuckii* NSD001 and *S. aureus* NSA739 strains were cultured to the mid-exponential growth phase (OD_600_ nm = 0.6–0.8) in TSB medium for osteoblast infection. After 5 min at 3220 g the collected bacteria were resuspended in sterile PBS. Osteoblasts (1x10^6^ cells/mL in 24-well plates) were infected with *S. debuckii* NSD001 or *S. aureus* NSA739 at a MOI of 100:1 (bacteria/cells) for 1h at 37°C and 5% CO_2_. After infection, the extracellular bacteria were killed with gentamicin (100 µg/mL) for 30 min. Following gentamicin treatment, osteoblasts were washed twice with PBS (T0) and then incubated for 5 and 24 h in fresh medium with 5 µg/mL of lysostaphin. Intracellular bacteria were counted at times 0, 5 and 24 h by lysing infected osteoblasts with 0.1% Triton X-100 in PBS. Osteoblast lysates were serially diluted on TSA plates and grown for 24 h at 37°C to determine the number of CFU.

### Cell viability assay

The release of lactate dehydrogenase (LDH) was measured using the MCE LDH Cytotoxicity Assay Kit (MedChemExpress USA). Cells were infected with a MOI of 20 for macrophages and 100 for osteoblasts, except that the cells were placed in a 96-well microplate and the extracellular bacteria were not eliminated. A microplate reader (Tecan, Model Spark, Grödig, Austria GmbH) was used to measure absorbance at 490 nm (A_490_ nm).

### Kinetics of biofilm formation

Biofilm formation was assessed using the Biofilm Ring Test^®^ technique (Biofilm Control, Saint-Beauzire, France) (Chavant et al., 2007). Bacterial cultures in Brain Heart Infusion (BHI) broth containing magnetic beads (TON004) were deposited on 96-well microplates (Falcon 96 Flat Bottom Transparent, Corning, USA) and incubated at 37°C without shaking. Medium and beads only were used as negative control. After incubation, 100 µL of liquid contrast solution (LIC001) was added into each well. Microplates were placed for 1 min on a magnetic block and then onto a reader (Epson Scanner modified for microplate reading). Image acquisitions were collected for each well after 1, 2, 3, 4 and 24 h of incubation, and analyzed using the BFC Elements 3.0 software (Biofilm Control) to obtain a Biofilm Formation Index (BFI). BFI values ranged from 0 for total bead immobilization (i.e., strong biofilm formation) to 20 for no bead aggregation (i.e., no biofilm formation).

### Zebrafish model and ethical statement

Zebrafish experiments were carried out at the University of Montpellier following European Union recommendations for the care and use of laboratory animals (https://ec.europa.eu/environment/chemicals/lab_animals/index_en.htm (accessed on 23 March 2024) and were approved by the Direction Sanitaire et Veterinaire de l’Herault and Comite d’Ethique pour l’Experimentation Animale under reference CEEA-LR-13007. Zebrafish were maintained, crossed, raised and staged as described previously (Kimmel et al., 1995; Westerfield, 2000). All studies were conducted on AB wild-type embryos prior to the free feeding stage. To prevent pigmentation and enhance optical transparency of embryos, 1X 1-phenyl 2-thiourea (PTU) were added to fish water at 24 h post-fertilization (hpf). At 30 hpf, embryos were dechorionated and anaesthetized with Tricaine (0.3 mg/mL) for microinjections.

### Injection of zebrafish embryos

Overnight cultures of *S. debuckii* NSD001 and *S. aureus* NSA739 in TSB were diluted in TSB and incubated at 37°C and 180 rpm until they reached OD_600 nm_ = 0.8. Cultures were centrifuged, and pellets were washed twice, then resuspended in PBS. Suspensions were homogenized through a 26-gauge needle and distributed into Eppendorf tubes before being stored at -80°C. For microinjections, an aliquot of each bacterium was thawed and diluted in PBS to obtain 4500 CFU/nL. At 35 hpf, 50 embryos per condition were infected by intravenous microinjection of 1 nL into the caudal vein and placed individually into 48-well plates in fish water. PBS injection was used as a negative control.

### Whole genome sequencing and analysis

*S. debuckii* NSD001 and *S. aureus* NSA739 were cultivated at 37°C for 24 h on Columbia sheep blood agar plates (5%) (Biomérieux). Genomic DNA was extracted using the DNeasy UltraClean Microbial Kit (Qiagen, Aarhus, Denmark) and eluted in a 50µL volume. For whole genome sequencing (WGS), the Illumina library preparation was performed using 250 ng of the extracted DNA following the DNA Prep kit library paired-end protocol (Illumina, San Diego, USA) and sequenced in a 39-h paired-end run providing 2x250-bp reads on a Miseq sequencer (Illumina). After sequencing data quality validation using FastqC software (version 0.23.2), NSD001 and NSA739 genomes were *de novo* assembled using Spades software (version 3.15.4), then aligned on Type-Strain Genomes Server (https://tygs.dsmz.de/user_requests/new) and RECOPHY (https://recophy.unibas.ch/recophy/) online platforms for reliable genome-based bacteria identification. Antimicrobial resistance encoding genes, virulence, pathogenicity, and plasmids were predicted using the CGE online platform (http://www.genomicepidemiology.org/services/). Genome annotation was performed on DDBJ Fast Annotation and Submission Tool online platform (https://dfast.ddbj.nig.ac.jp/). Pangenome analysis was performed on NSD001 and NSA739 annotated genomes using Roary tools (Version 3.13.0) available on Galaxy online software (https://www.usegalaxy.org.au/) to further identify virulence and biofilm genes.

### Statistical analyses

GraphPad Prism version 10.2.3 was used to perform statistical analyses, which are indicated in the relevant figure legends.

## Results

### Description of *Staphylococcus debuckii* clinical isolate

The patient was a 67-year-old man who was hospitalized at Nîmes University Hospital (France) due to sepsis and desaturation during chronic hemodialysis. The patient was a poorly controlled type 2 diabetic with Grade 1 obesity, hypertension, and a history of smoking. He had been undergoing treatment for stage Ib pulmonary adenocarcinoma (cT2sN0M0) for the past four months. Before one of his dialysis sessions, the patient presented with chills and a fever of 39°C. He was admitted to the Emergency Department at Nîmes University Hospital. Although the patient was afebrile (37.6°C) at admission, he had a necrotic lesion on the hallux of his left foot, Grade 3O following the IWGDF classification (Senneville et al., 2023), with suspicion of osteomyelitis due to a positive probe-to-bone test, signs of hallux necrosis, and inflammatory lymphangitis on the dorsum of the foot. Clinically, the necrosis was dry, peripheral pulses were absent, and there was no pain. No signs of septic shock were observed. His C-Reactive Protein level was 250 mg/L. After two days, an amputation of the hallux was carried out, and tissue and bone biopsies were performed by a trained surgeon simultaneously. The samples were immediately sent to the Department of Microbiology. The patient was then admitted to the Nephrology Unit. A probabilistic intravenous antimicrobial therapy regimen combining ertapenem and ofloxacin was initiated. After one week, bacterial cultures identified an ESBL-producing *E. coli* and *S. debuckii* in the samples. Ertapenem was discontinued, and a combination therapy of ofloxacin (400 mg per day) and cotrimoxazole (800 mg/160 mg per day) was continued for six weeks. After 40 days, the patient was transferred to a rehabilitation center. Unfortunately, he died two weeks later due to a general deterioration in his health.

### Growth and biofilm formation by *S. debuckii*

**Figure 1:**
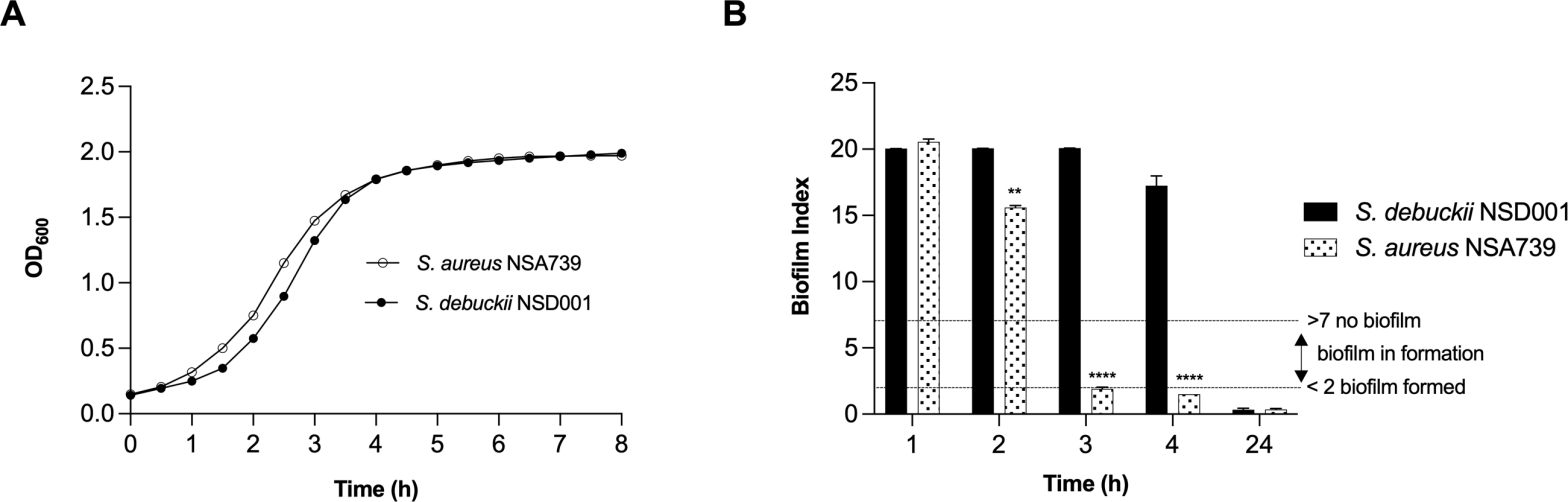
Planktonic growth and biofilm formation of *S. debuckii* NSD001. **(A)** The growth curves of *S. debuckii* NSD001 (black circles) and *S. aureus* NSA739 (white circles), cultured in Tryptic Soy Broth (TSB) for 24 h at 37°C with agitation at 108 rpm, display the mean optical density at 600 nm (OD_600_) with standard deviation (SD) from three biological replicates. **(B)** Biofilm formation kinetics of *S. debuckii* NSD001 and *S. aureus* NSA739 in Brain Heart Infusion (BHI), measured using the Biofilm Ring Test^®^. The Biofilm Index **(BI)** values, ranging from 0 to 20, classify the extent of biofilm development, with values under 2 indicating well-established biofilm and values over 7 indicating the absence of biofilm, as shown by the dotted horizontal lines. These values represent the mean ± SD derived from six separate experiments. (**p < 0.01; ****p < 0.0001; Two-way ANOVA test).

To investigate the pathogenic profile of the clinical *S. debuckii* isolate, we examined its growth and biofilm formation potential. In an *in vitro* setting, both *S. debuckii* NSD001 and *S. aureus* NSA739, a reference clinical strain isolated from a Grade 3 diabetic foot infection, exhibited typical bacterial growth (**Figure 1A**). During the exponential phase, no significant differences were observed in the growth rates of the two strains. The generation time for both, calculated using the formula G = ln(2)/μmax, was approximately 45 minutes, indicating similar growth dynamics (**Figure 1A**). Furthermore, we employed the Biofilm Ring Test^®^ technique to evaluate the biofilm-forming abilities of *S. debuckii* NSD001 and *S. aureus* NSA739. **Figure 1B** shows the profile of biofilm formation by *S. debuckii* NSD001 compared to *S. aureus* NSA739 over a 24 h period. In the initial stages, particularly at the 2 h interval, *S. debuckii* NSD001 demonstrates a discernible delay in biofilm formation, as evidenced by a significantly lower biofilm index than that of *S. aureus* NSA739. As the incubation period extends, *S. debuckii* NSD001 shows a progressive increase in biofilm formation, evidenced by ascending biofilm index values at the 3 h and 4 h intervals. By the 24 h time point, the biofilm formation for *S. debuckii* NSD001 has advanced considerably, culminating in a biofilm index approaching the levels observed for *S. aureus* NSA739 (**Figure 1B**). The convergence of the biofilm indices at the 24 h mark suggests that, despite an initial slower rate of biofilm formation, *S. debuckii* NSD001 possesses the ability to establish biofilm over an extended period, which could have implications for its persistence and virulence in host environments.

### *S. debuckii* persists within murine macrophages

**Figure 2:**
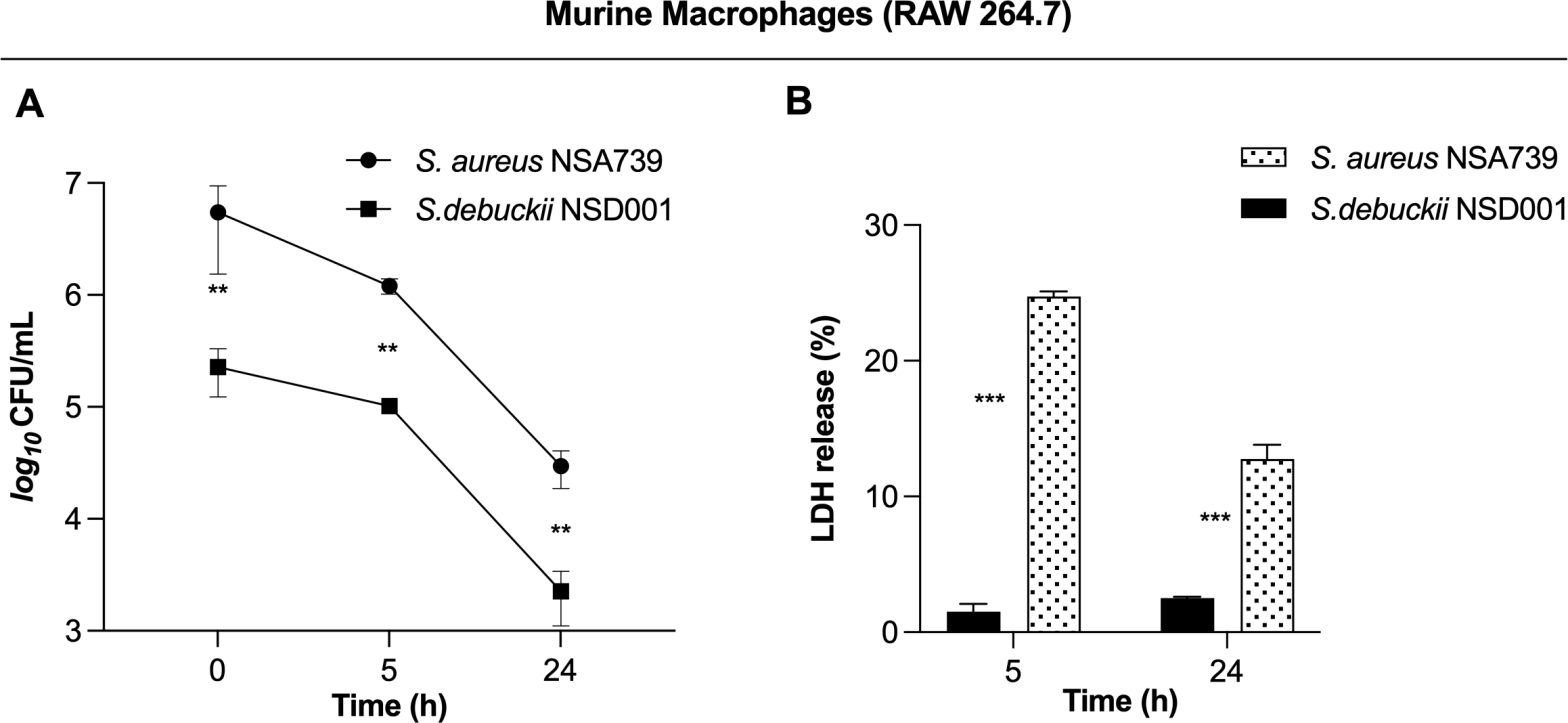
Survival of *S. debuckii* NSD001 in macrophages. RAW 264.7 macrophages were infected with *S. debuckii* NSD001 and *S. aureus* NSA739 at a multiplicity of infection (MOI) of 20 for 1 h at 37°C, followed by a 30-min incubation with gentamicin. Subsequently, the cells were incubated in complete media supplemented with lysostaphin to eliminate extracellular bacteria that might be released from lysed macrophages during the successive incubation time, and then lysed with 0.1% Triton X-100. **(A)** Macrophage cells were lysed, and surviving bacteria in lysates were evaluated by counting CFUs at 0, 5 and 24 h after lysostaphin/gentamicin treatment. **(B)** LDH release was measured using the MCE LDH Cytotoxicity kit after RAW 264.7 cells were infected at a MOI of 20 for 5 and 24 h, with the exception that the cells were seeded in a 96-well plate and the extracellular bacteria were not eliminated. The data represent the mean ± SD of five independent experiments. (**p < 0.01; ***p < 0.001; Two-way ANOVA test).

To investigate how *S. debuckii* interacts with macrophages, experiments were performed using the murine macrophage cell line RAW 264.7. Macrophages were allowed to undergo phagocytosis of either *S. debuckii* NSD001 or *S. aureus* NSA739, and viable bacteria were collected at 5 h and 24 h post-phagocytosis. Phagocytosis levels were similar between both strains, indicating comparable rates of engulfment by macrophages. Both strains were able to persist within macrophages without replication over the observed interval from 0 to 24 hpi, although the *S. debuckii* NSD001 strain showed lower persistence (**Figure 2A**). Moreover, the LDH assay that stands as a well-established means of identifying necrotic cell death revealed that upon infection with *S. aureus* NSA739, RAW264.7 macrophages showed a time-dependent increase in LDH release into the culture supernatants while infection with *S. debuckii* NSD001 resulted in consistently low levels of LDH release at both T5h and T24h, demonstrating a lack of cytotoxicity toward the RAW264.7 macrophages (**Figure 2B**). These observations suggest that unlike *S. aureus* NSA739, which shows a significant peak in cytotoxic activity at T5h followed by a decrease at T24h, *S. debuckii* NSD001 can survive and replicate within macrophages without causing host cell toxicity. The differential LDH release responses demonstrates the capacity of *S. debuckii* for non-cytotoxic survival within immune cells, raising significant considerations for the treatment of persistent intracellular infections.

### *S. debuckii* persists and replicates within human osteoblasts

**Figure 3:**
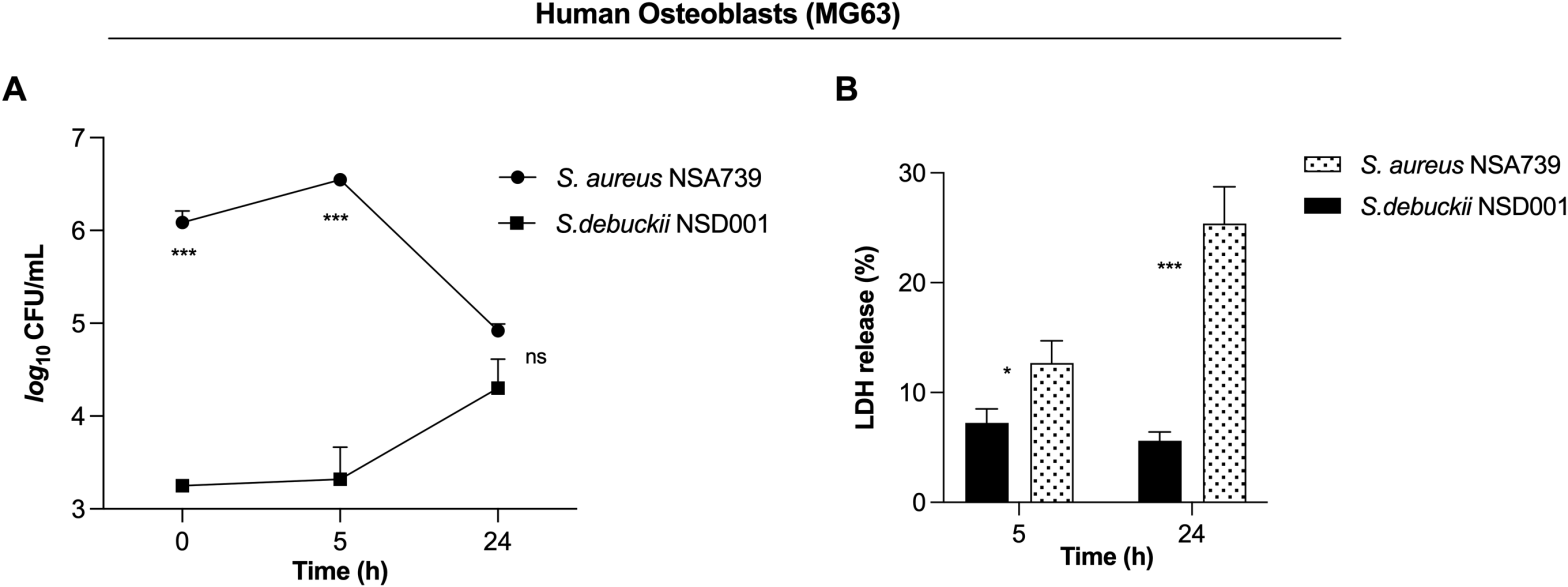
*S. debuckii* NSD001 replicates and persists in infected osteoblasts*. S. debuckii* NSD001 and *S. aureus* NSA739 were used to infect MG63 osteoblast cells at a multiplicity of infection (MOI) of 100 for 1h at 37°C, followed by a 30-min incubation with gentamicin. Subsequently, the cells were incubated in complete media supplemented with lysostaphin to eliminate extracellular bacteria that might be released from lysed cells during the successive incubation time, and then lysed with 0.1% Triton X-100. **(A)** Cells were lysed, and surviving bacteria in lysates were evaluated by counting CFUs at 0 (internalization), 5 and 24 h after lysostaphin/gentamicin treatment. **(B)** LDH release was measured using the MCE LDH Cytotoxicity assay kit after MG63 osteoblast cells were infected at a MOI of 100 for 5 and 24 h, with the exception that the cells were seeded in a 96-well plate and the extracellular bacteria were not eliminated. The data represent the mean ± SD of five independent experiments. (ns, not significant; *p < 0.05; ***p < 0.001; Two-way ANOVA test).

We identified the *S. debuckii* NSD001 strain in a case of DFOM, indicating its capability to infect and persist within non-phagocytic cells such as osteoblasts. The invasion of non-phagocytic host cells by *S. debuckii* could appear to be an effective strategy for evading elimination and sustaining infection. To test this hypothesis, infections were conducted using the human osteoblast cell line MG63 to assess the survival ability of *S. debuckii* NSD001 as previous studies have shown that *S. aureus* can proliferate and persist intracellularly in MG63 cells (Josse et al., 2015; Stracquadanio et al., 2021). Initial assays quantifying phagocytosis revealed that *S. debuckii* NSD001 was internalized by host cells at a significantly lower rate than *S. aureus* NSA739 (**Figure 3A**). Interestingly, while the intracellular load of *S. aureus* NSA739 remained stable at 5 h post-internalization and tended to decrease at 24 h, *S. debuckii* NSD001 started from a lower phagocytosis rate, but not only persisted but also increased its intracellular load at 24 h post-internalization (**Figure 3A**). In addition, we tested whether the increased survival of *S. debuckii* NSD001, could be associated with a decreased cytotoxicity of the infected osteoblast cells. When infected with *S. aureus* NSA739, MG63 cells exhibited a gradual increase in LDH release into the culture supernatants over time. Conversely, infection with *S. debuckii* NSD001 resulted in significantly decreased LDH levels (**Figure 3B**). These findings indicate that despite a low phagocytosis rate *S. debuckii* NSD001 can survive and replicate intracellularly in osteoblasts at a late-stage post-infection without inducing host cell toxicity. Therefore, the inability of osteoblasts to effectively eliminate intracellular *S. debuckii* NSD001 could serve as a substantial reservoir within cells, contributing significantly to the development and persistence of osteomyelitis.

### Investigating the virulence profile of *S. debuckii* NSD001 using a zebrafish embryo infection model

**Figure 4:**
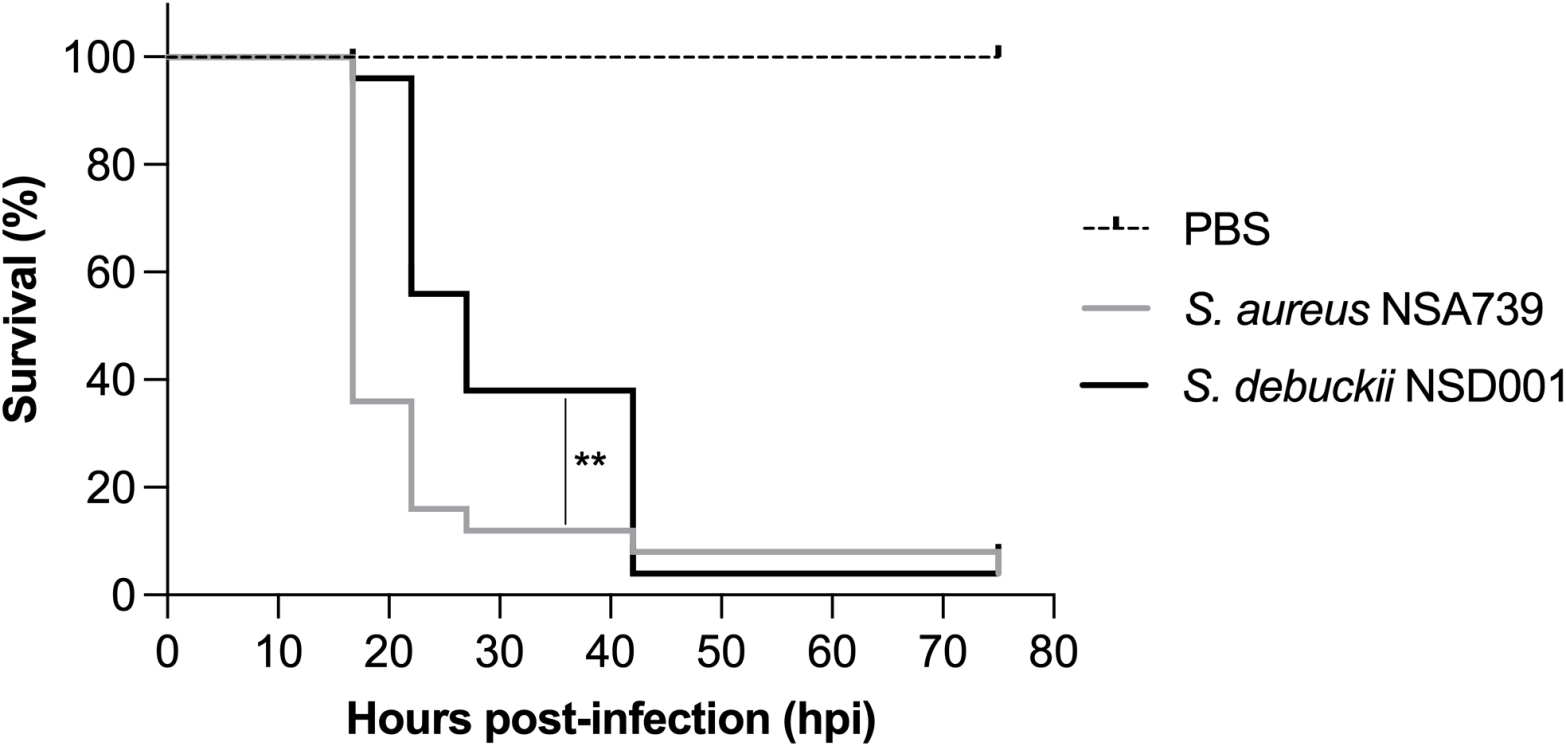
Virulence of *S. debuckii* NSD001 in zebrafish embryos. Kaplan–Meier survival plots illustrate the survival of zebrafish embryos following intravenous injection at 35 hpf with either *S. aureus* NSA739 (solid gray line) or *S. debuckii* NSD001 (solid black line) at a concentration of 4500 CFU/nL/embryo. Results are presented as the proportion of surviving embryos (n = 50 for each group, representing five separate experiments). Statistically significant differences in survival between the strains are indicated with asterisks: **p < 0.01 (log-rank test).

To evaluate the virulence of *S. debuckii* NSD001, bacterial strains were injected into the caudal vein of zebrafish embryos at 35 hours post-fertilization (hpf) as previously described (Prajsnar et al., 2008; Rasheed et al., 2021; Ahmad-Mansour et al., 2022). A pronounced and rapid decrease in the survival rate of embryos infected with *S. aureus* NSA739 is observed, with almost total lethality occurring as early as 20 hours post-infection (hpi), while *S. debuckii* NSD001 gradually decreases and culminates in the death of most embryos by 40 hpi (**Figure 4**). These findings indicate that despite a slower progression to lethality compared to *S. aureus* NSA739, the inevitability of mortality in all zebrafish embryos infected with *S. debuckii* NSD001 within 40 hpi clearly establishes its virulence.

### Comprehensive genomic analysis of *S. debuckii* NSD001

**Figure 5:**
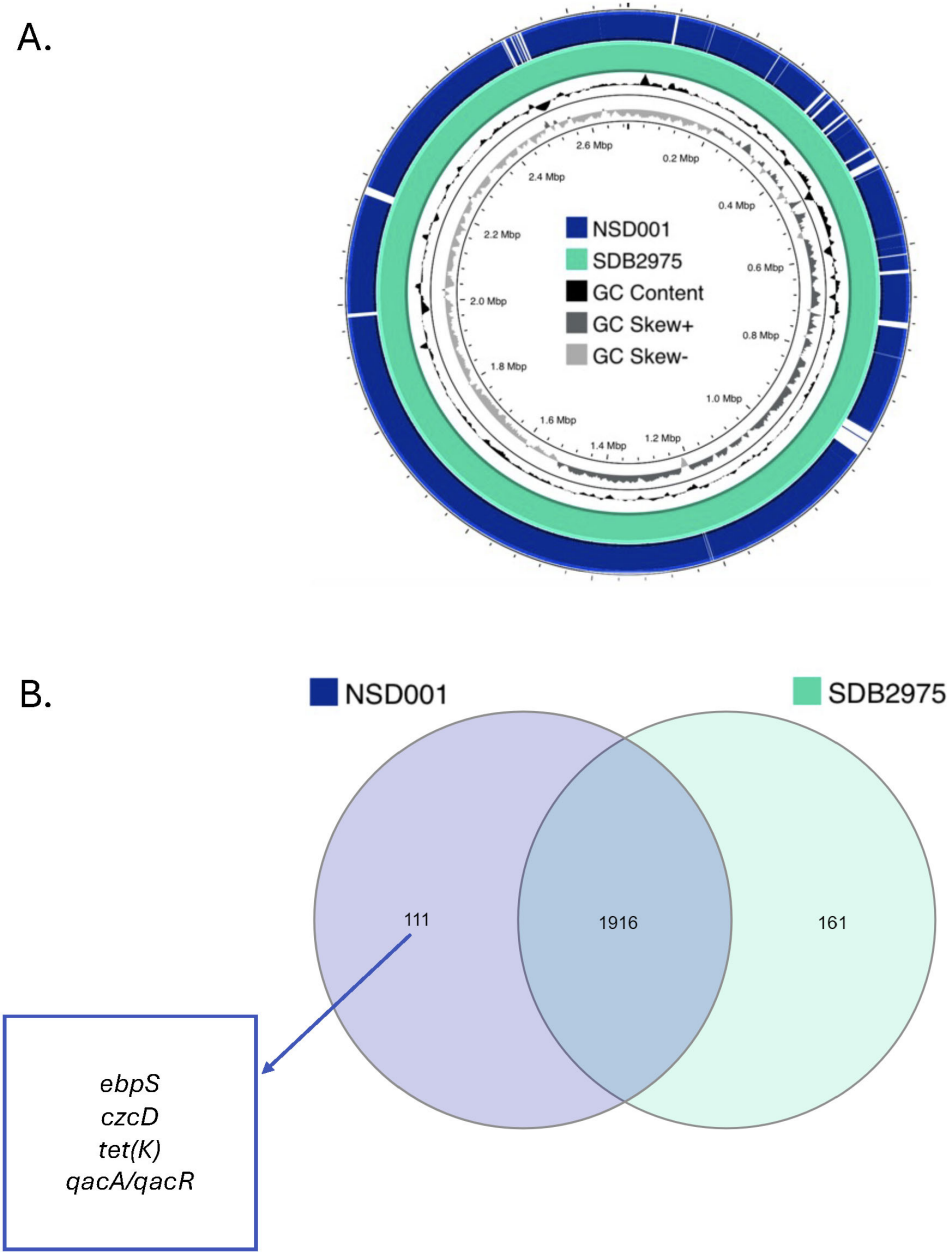
**(A)** Sequence comparison between the *S. debuckii* reference genome SDB2975 (light green) and the NSD001 genome (dark blue). Missing regions identified by BLAST analysis on the CGView server PROKSEE software are depicted as ‘gaps’ on the NSD001 genome. The inner ring corresponds to the GC skew of the *S. debuckii* NSD001 isolate. Dark gray profile indicates an overabundance of GC nucleotides, while light gray indicates the opposite. The inner black ring represents the GC%. **(B)** Venn Diagram showing shared and unique coding regions between *S. debuckii* strains SDB2975 and NSD001 after excluding genes encoding hypothetical proteins. Genes related to virulence and resistance are highlighted in the figure.

We sequenced the NSD001 genome to compare it with the *S. debuckii* reference genome SDB 2975. The NSD001 and SDB 2975 genome sizes are 2,576,588 bp and 2,691,850 bp, respectively (**Table 1**). Both *S. debuckii* genomes exhibited similar GC content, with values of 36.5% for NSD001 and 36.6% for SDB 2975. Genomic comparison between NSD001 and SDB2975 showed high identity and coverage, with 93.5% digital DNA-DNA hybridization (dDDH) and 90% identity (**Figure 5A**). **Figure 5B** presents a Venn diagram illustrating shared and unique coding regions between *S. debuckii* genomes SDB2975 and NSD001, excluding hypothetical protein-coding genes. Notably, NSD001 harbors unique genes linked to virulence and resistance, enhancing its potential for tissue adhesion (*ebpS*) and providing greater resistance to certain disinfectants and heavy metals (*qacA/qacR, czcD*). The resistome analysis of the NSD001 isolate corroborated the phenotypic antibiogram results, indicating resistance to tetracycline (*tetK*) (**Supplementary Table S1**). Additionally, multidrug efflux transporters (*norA*, *mdeA* and *sdrM* genes) were identified. Investigation of virulome content in NSD001 in comparison to strain NSA739 revealed distinct differences (**Supplementary Table S2**). Most of the toxins-encoding genes present in NSA739 isolate were absent in the NSD001 genome (*hlgABC, hld, lukE, lukD, sea, selX* and *sel26*). Instead, the detected virulence-encoding genes in NSD001 were primarily related to immune system evasion and invasiveness (e.g., *cap* genes, *eno*, and *katA*), lipoprotein maturation (*lspA* and *lgt*) and Clp proteases production (*clpB, clpL*, and *clpX*). Moreover, several two-component systems (TCS) involved in virulence regulation (*saeSR, vraSR* and *srrAB*) were identified in both NSD001 and NSA739 genomes, except for *nsaSR* in NSD001. Genes involved in the formation of the type IV pilus were present in both NSD001 and NSA739 genomes. Various well-known biofilm encoding genes factors were also identified in NSD001 (e.g., *aaa*, *sasA*, *sasF*, *ebps*, *clfB*, *clpP*, *psmβ, pmtABCD* operon), along with biofilm regulator-encoding genes (e.g., *agr* operon, *rsbUVW*-*sigB*, *lytSR*, *mgrA*, *cidA-lrgA*, *sarA*, *luxS mgrA*, *sarA*), which were also present in *S. aureus* NSA739 (**Supplementary Table S3**). Regarding the intracellular adhesion (*ica*) operon implicated in poly-Nacetylglucosamine (PNAG) production, only *icaC* and *icaR* genes were identified in NSD001, while *icaA*, *icaB*, and *icaD* were absent.

**Table 1:** Genome characteristics of NSD001, SDB2975, and NSA739 isolates.

	NSD001	SDB2975	NSA739
**Biosample**	SAMN40873641	SAMN07634888	SAMN39991533
**Bacterial species**	*Staphylococcus debuckii*	*Staphylococcus debuckii*	*Staphylococcus aureus*
**Genome size (bp)**	2,576,588	2,691,850	2,805,485
**GC content (%)**	36.5	36.6	32.7
**Number of CDSs**	2435	2583	2642
**Number of rRNAs**	5	18	7
**Number of tRNAs**	59	62	58
**Locality**	Nîmes (France)	Québec (Canada)	Nîmes (France)

ND, not determined; CDS, coding DNA sequences.

## Discussion

Our study represents the first detailed investigation into the virulence of *S. debuckii*, a recently identified pathogen. *S. debuckii* NSD001, isolated from a case of DFOM, provides critical insight into its pathogenic potential. Traditionally, CoNS like *S. debuckii* have been overshadowed by the clinical focus on more notorious pathogens such as *S. aureus*. However, the isolation of *S. debuckii* NSD001 from a human infection, transitioning from its initial discovery in bovine milk (Naushad et al., 2019), highlights a novel and concerning vector for human diseases, particularly in compromised hosts. This study not only establishes *S. debuckii* as a bona fide human pathogen but also challenges existing paradigms regarding the pathogenic potential of CoNS species, which is important, particularly for clinicians and treatment strategies (Michels et al., 2021). Our findings demonstrate the robust adaptability of *S. debuckii* NSD001, exhibiting capabilities for planktonic growth, biofilm formation, and persistence within phagocytic and non-phagocytic host cells, as well as virulence in the zebrafish model of infection.

We found no significant difference in planktonic growth rates between *S. debuckii* NSD001 and *S. aureus* NSA739, suggesting that both strains exhibit similar growth dynamics in a planktonic state. We identified biofilm-encoding genes in the *S. debuckii* NSD001 genome and confirmed its ability to form biofilm, accentuating its potential for chronic infection. Biofilms represent a significant hurdle in clinical settings, facilitating persistent infections that are notoriously resistant to antibiotics and immune clearance (Schilcher and Horswill, 2020; Durand et al., 2022). In the context of DFOM, where wounds are slow to heal, the presence of *S. debuckii* biofilms could lead to persistent infections, exacerbating the condition and potentially increasing the risk of amputation.

Moreover, *S. debuckii* NSD001 is significantly more susceptible to macrophage-mediated killing compared to *S. aureus* NSA739, as demonstrated by a greater than 1-log reduction in bacterial load within macrophages, while *S. aureus* NSA739 exhibits more aggressive evasion strategies, causing macrophage death 5 to 10 times faster than *S. debuckii*. This difference in survival dynamics suggests that while *S. debuckii* is more vulnerable to macrophage activity, it may avoid rapid immune destruction, allowing for a slower progression of infection. The fact that *S. debuckii* NSD001 is phagocytosed by macrophages at comparable rates than *S. aureus* NSA739 but shows lower persistence suggests a unique interaction with host immune cells. This could indicate an evasion mechanism that allows the bacteria to survive initial immune responses, potentially contributing to a more insidious and chronic infection process. This ability to avoid macrophage killing could facilitate the spread of the pathogen within the host, as recently demonstrated for another CoNS, *S. pettenkoferi* (Ahmad-Mansour et al., 2021; Magnan et al., 2022). Interestingly, in osteoblasts, *S. aureus* NSA739 demonstrates a lower intracellular survival compared to *S. debuckii* NSD001, which not only persists but also successfully replicates without inducing significant cytotoxic effects, indicating that *S. debuckii* NSD001 can invade and replicate within non-phagocytic cells such as osteoblasts, as already known for *S. aureus* (Claro et al., 2011; Musso et al., 2021; Stracquadanio et al., 2021). Moreover, as observed with *S. aureus* small colony variants (SCVs), which exhibit enhanced survival within osteoblasts despite a lower virulence profile (von Eiff et al., 1997), *S. debuckii* NSD001 demonstrates similar persistence within these bone cells, suggesting an adaptation that may contribute to chronic infection in bone-related environments. This is a significant observation as osteoblasts are central to bone health and integrity, and their infection can lead to amputation occurring in DFOM. The increased intracellular load of *S. debuckii* NSD001 over time in osteoblasts points to an adaptation for intracellular survival and proliferation, reminiscent of behaviors observed in facultative intracellular pathogens (Riffaud et al., 2023).

The virulence profile of *S. debuckii* NSD001 was also revealed through the zebrafish embryo infection model. *S. aureus* NSA739 demonstrates a swift and aggressive onset of lethality, underscoring its well-documented virulence. In contrast, *S. debuckii* NSD001 slower induction of mortality suggests a more surreptitious mode of pathogenesis. While *S. aureus* NSA739 is more pathogenic in the zebrafish model, the slower kill rate of *S. debuckii* NSD001 may suggest an adaptation to evade initial immune defenses, allowing it more time to establish infection. This could be a significant factor in clinical settings, where early detection and treatment are crucial.

In addition, the comprehensive genomic analysis of *S. debuckii* NSD001 not only delineates its genomic architecture but also elucidates its distinct virulence and resistance mechanisms, revealing insights into the strain pathogenicity. The rapid mortality of zebrafish embryos infected with *S. aureus* NSA739, in contrast to *S. debuckii* NSD001, can be attributed to the presence of hemolysin and enterotoxin genes present in NSA739, which are absent in NSD001. This absence likely diminishes NSD001 capacity for immediate cytotoxic effects. Additionally, the lack of some genes related to immune evasion and invasiveness, may explain the slower kill rate observed in embryos. NSD001 harbored genes that enhance its potential for tissue adhesion (*ebpS*) and resistance to antimicrobials and heavy metals (*qacA/qacR, czcD*), contributing to increased pathogenicity and environmental persistence. Furthermore, the presence of genes for competence type IV pilus assembly proteins in both *S. debuckii* NSD001 and *S. aureus* NSA739 suggests a potential role in enhancing the virulence of *S. debuckii*, as these structures facilitate genetic exchange and adaptability, key factors in bacterial pathogenicity (Muschiol et al., 2019).

In conclusion, *S. debuckii* NSD001 demonstrates a lower virulence profile than *S. aureus* NSA739, however, its ability to establish and persist within host tissues remains concerning. This comparatively reduced virulence may enable *S. debuckii* NSD001 to evade immune detection more effectively, allowing it to persist in host niches and potentially contribute to chronic infections. Unlike the acute pathogenicity observed with *S. aureus* NSA739, the persistence of *S. debuckii* NSD001 could pose a significant challenge in clinical settings, particularly in diabetic foot infections, where infections are harder to detect and treat. This capacity for sustained infection highlights the need for vigilance in monitoring *S. debuckii* NSD001 as an emerging CoNS pathogen in the context of diabetic foot infections. While this study focuses on the *S. debuckii* NSD001 strain due to its clinical relevance, a broader investigation across multiple *S. debuckii* isolates would be valuable to gain a more comprehensive understanding of its pathogenic mechanisms and to further characterize *S. debuckii* as an emerging pathogen.

## Data Availability

The datasets presented in this study can be found in online repositories. The names of the repository/repositories and accession number(s) can be found in the article/**Supplementary Material**.
