# Supplementary material for: Deciphering pathogenicity and virulence of the first Staphylococcus debuckii isolate from diabetic foot osteomyelitis
Source: Front Cell Infect Microbiol. 2024 Dec 17;14:1489280. doi: 10.3389/fcimb.2024.1489280 (PMC11685071; doi:10.3389/fcimb.2024.1489280)
Supplement: Supplementary file 1 [file Table1.docx]

**Table S1. Resistome analysis of *S. debuckii* NSD001 and *S. aureus* NSA739 strains**

| **Gene** | **Annotation** | **NSD001** | **NSA739** |
| --- | --- | --- | --- |
| *tet(K)* | Tetracycline efflux MFS transporter Tet(K) | WP_000492283.1 | - |
| *catA* | Type A-8 chloramphenicol O-acetyltransferase | - | WP_000143686.1 |
| *mecA* | PBP2a family beta-lactam-resistant peptidoglycan transpeptidase MecA | - | WP_001801873.1 |
| *qacA/qacR* | Quaternary ammonium compound efflux MFS transporter QacA/QacR | WP_000622776.1 WP_001832666.1 | - |
| *norA* | Multidrug efflux MFS transporter NorA | WP_001830321.1 | WP_001041274.1 |
| *norC* | Multidrug efflux MFS transporter NorC | - | WP_001006441.1 |
| *norB* | Multidrug efflux MFS transporter NorB | - | WP_000414695.1 |
| *mdeA* | Multidrug efflux MFS transporter MdeA | WP_011443659.1 | WP_011443659.1 |
| *mepA/R* | Multidrug efflux MATE transporter MepA and transcriptional repressor MepR | - | WP_000651051.1 WP_000397416.1 |
| *sdrM* | Multidrug efflux MFS transporter SdrM | WP_001237506.1 | WP_001237506.1 |
| *sepA* | Multidrug efflux transporter SepA | - | WP_000636857.1 |
| *lmrS* | Multidrug efflux MFS transporter LmrS | - | WP_001141007.1 |

**Table S2. Comparison of virulence-related genes between the *S. debuckii* NSD001 and *S. aureus* NSA739**

|  | | | **Putative Genetic Determinants of virulence** | | **NSD001** | **NSA739** |
| --- | --- | --- | --- | --- | --- | --- |
| **Toxins** | | | *hlgABC* | Haemolysin gamma A, B, and C | - | WP_000594519.1 WP_000783428.1 WP_000916704.1 |
|  |  |  | *hld* | Delta-hemolysin | - | WP_001549197.1 |
|  |  |  | *lukE, lukD* | Bi-component leukocidin LukED | - | WP_000473596.1 WP_000782463.1 |
|  |  |  | *sea* | Enterotoxin A | - | WP_000475325.1 |
|  |  |  | *selX* | Staphylococcal enterotoxin-like toxin X | - | WP_000475325.1 |
|  |  |  | *sel26* | Staphylococcal enterotoxin type 26 | - | WP_000889280.1 |
| **Avoid Host Immune Response and Invasiveness** |  | | *capA* | Capsular polysaccharide type 5/8 biosynthesis protein CapA | WP_000446299.1 | WP_000446299.1 |
|  |  | | *cap8B* | Type 8 capsular polysaccharide synthesis protein Cap8B | WP_000037332.1 | WP_000037332.1 |
|  |  | | *cap8C* | Type 8 capsular polysaccharide synthesis protein Cap8C | - | WP_000565304.1 |
|  |  | | *cap8D* | Type 8 capsular polysaccharide synthesis protein Cap8D | WP_000940790.1 | WP_000940790.1 |
|  |  | | *cap8E* | Type 8 capsular polysaccharide synthesis protein Cap8E | WP_000459062.1 | WP_000459062.1 |
|  |  | | *cap8F* | Type 8 capsular polysaccharide synthesis protein Cap8F | WP_001028283.1 | WP_001028283.1 |
|  |  | | *cap8G* | Type 8 capsular polysaccharide synthesis protein Cap8G | WP_000413171.1 | WP_000413171.1 |
|  |  | | *cap8L* | Type 8 capsular polysaccharide synthesis protein Cap8L | - | WP_001291020.1 |
|  |  | | *cap8M* | Type 8 capsular polysaccharide synthesis protein Cap8M | - | WP_000825098.1 |
|  |  | | *cap8O* | Type 8 capsular polysaccharide synthesis protein Cap8O | - | WP_000723436.1 |
|  |  | | *cap8P* | Type 8 capsular polysaccharide synthesis protein Cap8P | - | WP_000723436.1 |
|  |  | | *eno* | Enolase | WP_001829595.1 | WP_001121760.1 |
|  |  | | *katA* | Catalase A | WP_000082539.1 | WP_000082539.1 |
|  |  | | *tuf* | Elongation factor tu | WP_001832289.1 | WP_001040568.1 |
|  |  | | *lspA* | Lipoprotein signal peptidase | WP_002494957.1 | WP_000549207.1 |
|  |  | | *lgt* | Lipoprotein diacylglycerol transferase | WP_000513305.1 | WP_000513305.1 |
|  |  | | *nuc* | Thermonuclease | - | WP_000141557.1 |
|  |  | | *coA* | Staphylocoagulase | - | WP_000744096.1 |
|  |  | | *spA* | *Staphylococcus* protein A | - | WP_000728765.1 |
|  |  | | *vwb* | von Willebrand factor binding protein Vwb | - | WP_000791702.1 |
|  |  | | *isaB* | Immunodominant staphylococcal antigen IsaB | - | WP_001044560.1 |
|  |  | | *scn* | Staphylococcal complement inhibitor | - | WP_000702263.1 |
|  |  | | *sak* | Staphylokinase | - | WP_000920038.1 |
| **Exoenzymes, Protease activity or implicated in proteolysis** | |  | *aur* | Aureolysin | - | WP_001821522.1 |
|  |  |  | *splA, splB, splE* | Serine proteases SplA, SplB, SplE | - | WP_001039427.1  WP_001039454.1  WP_001038872.1 |
|  |  |  | *clpB, clpL, clpX* | Clp proteases | WP_000353954.1 WP_001058993.1 WP_001830765.1 | WP_000353954.1 WP_001058993.1 WP_000472302.1 |
|  | |  | *mecA* | Adapter protein MecA | WP_001829308.1 | WP_001217728.1 |
| **Regulation** | | | *nsaSR* | Sensor histidine kinase/DNA-binding response regulator | - | WP_000143425.1 WP_000697877.1 |
|  |  |  | *srrAB* | Transcriptional regulatory protein SrrA / Sensor protein SrrB | WP_001831066.1 WP_000987774.1 | WP_000064078.1 WP_000987774.1 |
|  |  |  | *vraSR* | Sensor Protein VraS/ Response regulator protein VraR | WP_001017131.1 WP_000153535.1 | WP_001017131.1 WP_000153535.1 |
|  |  |  | *saeSR* | Histidine protein kinase SaeS /Response regulator SaeR | WP_000244415.1 WP_000149344.1 | WP_000244415.1 WP_000149344.1 |
| **Others** | | | *cvfB* | Conserved virulence factor B | WP_001162352.1 | WP_001162352.1 |
|  |  |  | *comGA,* | Competence type IV pilus ATPase ComGA | WP_001831109.1 | WP_000697220.1 |
|  |  |  | *comGB* | Competence type IV pilus assembly protein ComGB | WP_001831131.1 | WP_000776422.1 |
|  |  |  | *comGC* | Competence type IV pilus major pilin ComGC | WP_001831271.1 | WP_000472256.1 |
|  |  |  | *comGD* | Competence type IV pilus minor pilin ComGD | WP_001788899.1 | WP_001788899.1 |
|  |  |  | *comGE* | Competence protein ComGE | YP_005742247.1 | WP_011447021.1 |
|  |  |  | *comGF* | Competence type IV pilus minor pilin ComGF | WP_002440043.1 | WP_001788897.1 |
|  |  |  | *comGG* | Competence protein ComGG | - | - |

**Table S3. Comparison of biofilm-related genes between *S. debuckii* NSD001 and *S. aureus* NSA739**

| **Biofilm steps** | **Putative Genetic Determinants of biofilm production** | | **NSD001** | **NSA739** |
| --- | --- | --- | --- | --- |
| **ADHESION** | *aaa* | Autolysin/adhesin Aaa | WP_001170264.1 | WP_001170264.1 |
|  | *sasC* | LPXTG-anchored repetitive surface protein SasC | - | WP_001050566.1 |
|  | *sasA* | Serine-rich repeat glycoprotein adhesin SasA | WP_000044547.1 | WP_000044547.1 |
|  | *sasF* | Cell-wall-anchored protein SasF | WP_001151890.1 | WP_001151890.1 |
|  | *ebpS* | Elastin-binding protein EbpS | WP_107515759.1 | WP_000069289.1 |
|  | *emp* | Extracellular matrix protein-binding adhesin Emp | - | WP_000728068.1 |
|  | *fnbA, fnbB* | Fibronectin binding proteins A and B | - | WP_000794614.1 WP_000841404.1 |
|  | *clfA* | Clumping factor A | - | WP_001056195.1 |
|  | *clfB* | Clumping factor B | WP_000745891.1 | WP_000745891.1 |
|  | *sdrC, sdrD, sdrE* | Serine-aspartate repeat-containing proteins C, D and E | - | WP_001060462.1 WP_000934467.1 WP_000610259.1 |
| **MATURATION** | *aap* | Accumulation-associated protein Aap | - | WP_011082576.1 |
|  | *icaA* | Poly-beta-1,6 N-acetyl-D-glucosamine synthase IcaA | - | WP_001159430.1 |
|  | *icaD* | Intracellular adhesion protein IcaD | - | WP_000240580.1 |
|  | *icaB* | Intercellular adhesin biosynthesis polysaccharide N-deacetylase | - | WP_000877369.1 |
|  | *icaC* | polysaccharide intercellular adhesin biosynthesis/export protein IcaC | WP_000723833.1 | WP_000723836.1 |
|  | *isdA, isdB* | Iron-regulated surface determinant protein A, B | - | WP_000160859.1 WP_001041583.1 |
|  | *splA, splB,splC, splD,splF* | Serine proteases SplA, SplB, SplC, SplD, SplE | - | WP_001039427.1 WP_001039454.1 WP_001038872.1  WP_001038704.1 WP_001038752.1 |
|  | *clpP* | ClpP protease | WP_001829659.1 | WP_225426975.1 WP_001049165.1  WP_000642728.1 |
| **REGULATION** | *icaR* | Biofilm operon icaADBC regulator | WP_000653261.1 | WP_000653261.1 |
|  | agr operon *(agrBDCA)* | Accessory gene regulators A, B, C and D | WP_001830005.1 WP_047132797.1 WP_000447888.1 WP_000688492.1 | WP_001105696.1 WP_001093929.1 WP_000447888.1 WP_000688492.1 |
|  | *sigB* | Sigma B | WP_001041111.1 | WP_001041111.1 |
|  | *rsbUVW* | / | WP_001829902.1  WP_001829952.1  WP_001829903.1 | WP_000390829.1  WP_001052491.1 WP_011447044.1 |
|  | *lytSR* | LytSR two-component regulatory system | WP_002484941.1 WP_001831505.1 | WP_000925394.1 WP_000645452.1 |
|  | *arlRS* | ArlRS two-component system | - | WP_000192137.1  WP_000166801.1 |
|  | *mgrA* | HTH-type transcriptional regulator MgrA | WP_001830335.1 | WP_001283444.1 |
|  | *cidA* | Holin-like protein CidA | WP_001832353.1 | WP_000549734.1 |
|  | *lrgA* | Antiholin-like protein LrgA | WP_002469559.1 | WP_001792906.1 |
|  | *sarA* | Transcriptional regulator SarA | WP_001018677.1 | WP_001018677.1 |
|  | *luxS* | S-ribosylhomocysteine lyase | WP_001829929.1 | WP_000164421.1 |
| **DISPERSION** | *psmβ* | Beta-class phenol-soluble modulins | WP_000147103.1  WP_000398672.1 | WP_000147103.1  WP_000398672.1 |
|  | *pmtABCD* operon | Phenol-soluble modulin transporter Pmt | WP_001830432.1  WP_001830379.1 WP_001830444.1 WP_123145070.1 | WP_000991306.1 WP_001221651.1 WP_000763043.1 WP_000645727.1 |
